# Novel Norovirus in Dogs with Diarrhea

**DOI:** 10.3201/eid1606.091861

**Published:** 2010-06

**Authors:** João Rodrigo Mesquita, Leslie Barclay, Maria São José Nascimento, Jan Vinjé

**Affiliations:** University of Porto, Porto, Portugal (J.R. Mesquita, M.S.J. Nascimento); Polytechnic Institute of Viseu, Viseu, Portugal (J. R. Mesquita); Centers for Disease Control and Prevention, Atlanta, Georgia, USA (L. Barclay, J. Vinjé)

**Keywords:** Norovirus, canine, genogroup, dogs, viruses, zoonoses, dispatch

## Abstract

To identify the prevalence and genetic variability of noroviruses in dogs, we tested fecal samples by using reverse transcription–PCR. We found canine norovirus in 40% and 9% of dogs with and without diarrhea, respectively. The virus was genetically unrelated to other noroviruses and constitutes a tentative new genogroup.

Human noroviruses (NoVs) are the most frequent cause of epidemic and sporadic acute gastroenteritis worldwide among humans of all ages (*1*,*2*). The virus is transmitted through ingestion of contaminated food or water or from person to person through the fecal–oral route. The close genetic relatedness of swine NoV with human NoVs of genogroup (G) II suggests the potential for transfer from animals to humans (*3*–*5*). In addition, recent findings of viruses genetically related to human NoVs, as well as to animal NoV sequences in pigs and calves, have raised concerns about the possible emergence of recombinant viruses (*4*).

NoVs are genetically heterogeneous viruses that belong to the family *Caliciviridae*. The viral capsid encloses a single-stranded, positive-sense RNA genome of 7.3–7.7 kb that is organized in 3 open reading frames (ORFs), of which ORF1 encodes a polyprotein that is proteolytically cleaved into 6 nonstructural proteins, including RNA-dependent RNA polymerase (RdRp), helicase, and protease (*1*). ORF2 and ORF3 encode major (viral protein [VP] 1) and minor (VP2) capsid proteins. The GLPSG and YGDD motifs of the RdRp protein are conserved among all members of the family *Caliciviridae* (*6*). The genus *Norovirus* currently comprises 5 genogroups, designated GI–GV, which can be grouped into at least 32 genetic clusters (*2*,*7*). Only viruses from GI, GII, and GIV have been associated with human disease. Recently, the finding of a novel GIV norovirus in a young dog (*5*) indicated that pets can be infected with NoVs. To identify the prevalence and genetic variability of NoVs in dogs, we tested fecal specimens from dogs with and without diarrhea.

## The Study

A total of 105 fecal samples from dogs in municipal dog shelters, veterinary clinics, and pet shops from 3 districts (Porto, Viseu, and Guarda) in Portugal were collected during December 2007–November 2008. Veterinarians evaluated the dogs for diarrhea at the time of the visit. The fecal panel consisted of 63 samples from dogs with diarrhea and 42 samples from dogs with formed, normal brown feces (i.e., controls). All samples were kept at –20°C until processed. Fecal suspensions (10%) were made in phosphate-buffered saline pH 7.2, and solids were removed by centrifugation at 8,000 × *g* for 5 min. Nucleic acid was extracted by using the QIAamp viral RNA mini kit (QIAGEN, Hilden, Germany) according to the manufacturer's instructions and tested for the presence of NoV RNA by 2 broadly reactive NoV conventional reverse transcription–PCR (RT-PCR) assays (*8*,*9*), selective for a partial RdRp region of the genome, by using One-Step RT-PCR kit (QIAGEN) and 37°C as annealing temperature.

Two (2%) of the 105 fecal samples tested positive for NoV with primer pair JV12y/13i, whereas no RT-PCR products were found using the p289-p290 primer pair. The 2 JV12y/13i sequences were identical and contained the GLPSG amino acid motif characteristic of viral RNA polymerases.

We designed specific canine norovirus oligonucleotide primers JV102 (5′-TGG GAT TCA ACA CAG CAG AG-3′) and JV103 (5′-TGC GCA ATA GAG TTG ACC TG-3′) and retested all 105 samples. Fecal samples from 25 (40%) of the 63 dogs with diarrhea and 4 (9%) of the 42 controls tested positive for a new canine NoV (Viseu strain) (Table). An ≈3.3-kb fragment from the RdRp region in ORF1 to the poly-A tail, including the complete ORF2 and ORF3 genes, was generated by long-template RT-PCR by using previously described methods (*10*). Gel-purified PCR products were cloned and sequenced by primer walking, and a 3,357-nt sequence including partial ORF1, full-length ORF2 and ORF3, and the 3′ end noncoding region from the Viseu strain was obtained. The consensus sequence from 5 different clones was submitted to GenBank and assigned accession no. GQ443611. All 105 nucleic acid extracts also were tested by RT-PCR for the recently reported canine NoV strain (*5*), canine coronavirus (CCV) (*11*), and by PCR for canine parvovirus (CPV-2) combining oligonucleotide primers for the detection of types 2a and 2b (*12*). None of the samples tested positive for the reported GIV.2 canine NoV strain (GIV.2/170/07/ITA). A total of 32 (51%) samples from dogs with diarrhea tested positive for CCV, whereas 2 (5%) of the control samples were positive. In addition, 58 (55%) samples tested positive for CPV-2, of which 18 also were positive for the novel canine NoV (Table). Univariate analysis (SPSS version 17.0, SPSS Inc., Chicago, IL, USA) demonstrated that the novel canine NoV and CCV were significantly associated with diarrhea (Table).

**Table:** Detection of canine norovirus, parvovirus, and coronavirus in 105 fecal samples from dogs with and without diarrhea, Porto, Viseu, and Guarda, Portugal, December 2007–November 2008*

Diarrhea	Canine norovirus,† no. (%) dogs	Canine parvovirus 2,‡ no. (%) dogs	Canine coronavirus,§ no. (%) dogs	Negative, no. (%) dogs	Total no. dogs
Yes	25 (40)	36 (57)	32 (51)	11 (17)	63
No	4 (9)	22 (35)	2 (5)	18 (43)	42

*Detection by reverse transcription–PCR. †Odds ratio (OR) 6.25, 95% confidence interval (CI) 1.87–26.65; p = 0.0007 (univariate analysis). ‡OR 20.8, 95% CI 5.06–83.92; p<0.0001 (univariate analysis). §OR 1.21, 95% CI 0.51–2.86; p = 0.63 (univariate analysis).

## Conclusions

We detected novel norovirus sequences in 40% of samples from dogs with diarrhea and 9% of specimens from dogs without diarrhea. All canine norovirus RdRp sequences had a high nucleotide sequence identity (range 98%–100%) and differed 19%–22% from recently reported canine norovirus strains (*5*,*13*) (Figure, panel A).

**Figure:**
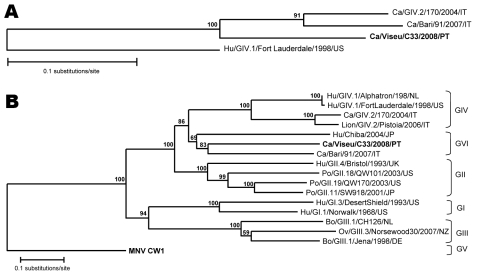
Phylogenetic trees of A) a 206-nt region of the RNA-dependent polymerase gene of 1 human genogroup (G) IV strain (Hu/GIV.1/FortLauderdale/1998/US), 2 recently published canine noroviruses (GIV.2/170/2004/IT, Bari/91/2007/IT) (*5*,*13*), and the novel canine Viseu strain reported in the study (**boldface**); and B) full-length amino acid sequence of viral protein (VP) 1 of norovirus strains of GI–GV detected in animals and human strains Hu/GI.1/Norwalk/1968/US, Hu/GI.3/DesertShield/1993/US, and Hu/GII.4/Bristol/1993/UK. The Viseu strain (**boldface**) forms tentatively a novel genogroup (GVI) with strains Ca/Bari/91/2007/IT and Hu/Chiba/2004/JP. Phylogenetic analysis was performed by using TreeCon software with Jukes and Cantor correction with bootstrap analysis (n = 1,000), and the tree topology was inferred by using neighbor-joining. Ca, canine; Hu, human; Po, porcine; Bo, bovine; Ov, ovine; Mu, murine.

In the complete VP1, the Viseu strain was most closely related to canine NoV strain Bari/91/07/IT (63.2% amino acid identity) and human strain Chiba/040502/2004/JP (55.1% identity) (Figure, panel B). Therefore, we tentatively classifed the Viseu strain, together with the Bari and Chiba strains, as a novel genogroup (GVI). To elucidate the potential pathogenic enteric role of the new canine NoV, we also tested all fecal specimens for CPV-2 and CCV, which are well-established enteric pathogens of dogs. Our results show that the novel canine NoV and CCV, but not CPV-2, were significantly associated with diarrhea.

For several reasons, our findings should be interpreted with caution. Viral shedding from dogs without diarrhea could represent asymptomatic infection, a resolution stage of the disease, or detection of CPV-2 vaccine virus. In addition, other canine enteric pathogens for which we did not test could bias the results. Further research that includes electron microscopy or infectivity studies is needed to confirm the existence of this new canine NoV.

The new virus sequences were detected in dogs from several dog owners throughout central Portugal and from a dog shelter in Viseu. Because Portuguese law requires official shelters to provide sanctuary for unwanted animals and keep them as adoptable pets for a certain period, humans potentially could be exposed to canine NoV that could increase the possibility for the emergence of canine/human NoV recombinants and increase the likelihood of zoonotic transmission. Additional serologic studies are needed to determine the level of exposure to canine NoVs in dogs and humans similar to previous studies on bovine NoV (*14*). Zoonotic transmission between dogs and humans is not new, and the close and often intimate interactions between these 2 species have been suggested as a major disease risk for humans (*15*). Only a few studies on NoV infections in dogs have been reported (*5*,*13*). Experimental infections in gnotobiotic dogs could ultimately provide essential data for describing the prevalence of canine NoVs in dogs and their causal relationship with diarrhea.
